# Assessment of Executive Function in Everyday Life—Psychometric Properties of the Norwegian Adaptation of the Children’s Cooking Task

**DOI:** 10.3389/fnhum.2021.761755

**Published:** 2022-02-03

**Authors:** Torun G. Finnanger, Stein Andersson, Mathilde Chevignard, Gøril O. Johansen, Anne E. Brandt, Ruth E. Hypher, Kari Risnes, Torstein B. Rø, Jan Stubberud

**Affiliations:** ^1^Children’s Clinic, St. Olav’s Hospital, Trondheim University Hospital, Trondheim, Norway; ^2^Department of Psychology, University of Oslo, Oslo, Norway; ^3^Rehabilitation Department for Children with Acquired Neurological Injury, Saint Maurice Hospitals, Saint Maurice, France; ^4^Sorbonne Université, Laboratoire d’Imagerie Biomédicale (LIB) Inserm, CNRS, Paris, France; ^5^Sorbonne Université, GRC 24 Handicap Moteur et Cognitif et Réadaptation (HaMCRe), Paris, France; ^6^Department of Clinical and Molecular Medicine, Norwegian University of Science and Technology, Trondheim, Norway; ^7^Department of Clinical Neurosciences for Children, Division of Pediatric and Adolescent Medicine, Oslo University Hospital, Oslo, Norway; ^8^Department of Research, Innovation and Education, Clinical Research Unit, St. Olav’s University Hospital, Trondheim, Norway; ^9^Department of Research, Lovisenberg Diaconal Hospital, Oslo, Norway

**Keywords:** executive function, child, adolescent, ecological assessment, ecological validity, psychometric properties, acquired brain injury, cognitive outcome

## Abstract

**Background**: There are few standardized measures available to assess executive function (EF) in a naturalistic setting for children. The Children’s Cooking Task (CCT) is a complex test that has been specifically developed to assess EF in a standardized open-ended environment (cooking). The aim of the present study was to evaluate the internal consistency, inter-rater reliability, sensitivity and specificity, and also convergent and divergent validity of the Norwegian version of CCT among children with pediatric Acquired Brain Injury (pABI) and healthy controls (HCs).

**Methods**: The present study has a cross-sectional design, based on baseline data derived from a multicenter RCT. Seventy-five children with pABI from two university hospitals with parent-reported executive dysfunction and minimum of 12 months since injury/completed cancer therapy, as well as 59 HCs aged 10–17 years, were assessed with CCT using total errors as the main outcome measure. The pABI group completed tests assessing EF (i.e., inhibition, cognitive flexibility, working memory, and planning) on the impairment level within the ICF framework (performance-based neuropsychological tests and the Behavioral Assessment of the Dysexecutive Syndrome for Children), and on the participation level (questionnaires). In addition, they completed tests of intellectual ability, processing speed, attention, learning, and memory. Finally, overall functional outcome (pediatric Glasgow Outcome Scale-Extended) was evaluated for the children with pABI.

**Results**: Acceptable internal consistency and good inter-rater reliability were found for the CCT. Children with pABI performed significantly worse on the CCT than the HCs. The CCT identified group membership, but the sensitivity and specificity were overall classified as poor. Convergent validity was demonstrated by associations between the CCT and performance-based tests assessing inhibition, cognitive flexibility, and working memory, as well as teacher-reported executive dysfunction (questionnaires). Divergent validity was supported by the lack of association with performance-based measures of learning and memory, attention, and verbal intellectual ability. However, there was a moderate association between the CCT and performance-based tests of processing speed. Lastly, better performance on the CCT was associated with a better functional outcome.

**Conclusion**: Our study with a relatively large sample of children with pABI and HC’s demonstrated good psychometric properties of the CCT. CCT performance was associated with the overall level of disability and function, suggesting that CCT is related to the level of activity in everyday life and participation in society. Hence, our study suggests that the CCT has the potential to advance the assessment of EF by providing a valid analysis of real-world performance. Nevertheless, further research is needed on larger samples, focusing on predictors of task performance, and evaluating the ability of CCT to detect improvement in EF over time. The patterns of error and problem-solving strategies evaluated by the CCT could be used to inform neuropsychological rehabilitation treatmentand represent a more valid outcome measure of rehabilitation interventions.

## Introduction

Self-control is imperative for optimal functioning in everyday life (Moffitt et al., 2011), and is one aspect of executive function (EF) which is often impaired after an acquired brain injury sustained during childhood (Beauchamp et al., 2011; Araujo et al., 2017). Pediatric acquired brain injuries (pABIs), either caused by traumatic brain injury (TBI) or non-traumatic insults (e.g., brain tumor, cerebrovascular accident, infection, and inflammation) are leading causes of childhood morbidity, mortality (World Health Organization, 2009), and acquired disability (Thurman, 2016). More severe injury and younger age at injury have been related to worse outcomes (Resch et al., 2019). Because pediatric ABI (pABI) affects a developing brain, the consequences go beyond the immediate brain injury, affecting social, emotional, and behavioral functioning, in addition to cognition (Brinkman et al., 2018; Resch et al., 2019). The International Classification of Functioning, Disability, and Health (ICF) provides a framework for describing and organizing information about a person’s function and disability after a pABI (World Health Organization, 2001), covering various levels of functioning and disability such as: (1) impairments in body function or structure; (2) limitations in activity; and (3) restriction of participation.

Among cognitive deficits, EF impairments are some of the most common and disturbing cognitive symptoms following pABI, affecting most domains of everyday life (Anderson et al., 2000; Anderson and Catroppa, 2005, 2006; Catroppa and Anderson, 2009; Catroppa et al., 2009; Stuss, 2011; Karver et al., 2012; Beauchamp and Anderson, 2013; Li and Liu, 2013; Ponsford et al., 2013; Michaeli et al., 2014; Ryan et al., 2014). EFs refer to a number of higher-order top-down processes enabling the individual to regulate, modulate and control thoughts, behavior, and emotion in a goal-directed manner (Cicerone et al., 2006; Friedman and Miyake, 2017). EFs enable a person to perform complex and novel tasks and cope with new problems that arise (Hughes and Graham, 2002). EFs are therefore essential for independence in activities of everyday life, as well as successful school and work attainment. However, EF is poorly defined (Baggetta and Alexander, 2016; Karr et al., 2018), with several theories attempting to conceptualize the different interrelated functions or processes that are included in the construct. For example, the Supervisory Attentional System (SAS) has been proposed to be applied in novel situations to avoid errors in goal achievement (Norman and Shallice, 1986). Some argue that the ability to actively maintain task goals and goal-related information and use this information to direct non-executive cognitive processes is a common underlying ability that affects all the other EFs (common EF; Miyake et al., 2000; Miyake and Friedman, 2012; Friedman and Miyake, 2017). The SAS, constituting the executive control of non-executive processes, has been proposed to be fractionated into different subprocesses (Shallice and Burgess, 1996; Shallice and Cooper, 2011). According to Miyake and Friedman (2012), EF is comprised of three interrelated, separable processes: *inhibition*, *working memory updating*, and *task-set shifting*. These functions are also reflected in Diamond’s model where EF is described as three interrelated cognitive processes: (a) *working memory* (the ability to hold information in mind and manipulate it); (b) *cognitive flexibility* (the ability to flexibly switch perspectives, focus of attention or response mappings); and (c) *inhibition* (the ability to ignore distraction and stay focused, and suppress or resist pre-potent responses; Diamond, 2006). Other models divide EF into *metacognitive EF* which includes problem- solving, planning, strategy development, and abstraction in addition to working memory, and *emotional/motivational EF* which includes the coordination and integration of cognition and emotion, as well as inhibitory control, emotional and behavioral regulation (Stuss and Knight, 2013). To add to this complexity, executive components are also included in models of attention (Petersen and Posner, 2012).

Although EF emerges early in childhood, the development of the different EF processes has been proposed to follow somewhat independent trajectories at various rates throughout childhood, adolescence, and young adulthood (Anderson, 2001; Best and Miller, 2010), with the transition from childhood to adolescence and young adulthood involving increasing demands on EF (Crone and Dahl, 2012). It has been suggested that EF abilities gradually differentiate from a unitary process in young children into a set of diverse, interactive processes from early school-age throughout adolescence and young adulthood (Miyake et al., 2000; Best and Miller, 2010; Karr et al., 2018). Confirmatory factor analysis has been applied to evaluate the factor structure of EF among school- aged children and adolescents. Initial analyses supported a three-factor model suggesting shifting/cognitive flexibility developing during middle childhood and adolescence (Karr et al., 2018). However, re-analyses suggested greater reliance on unitary EF within this group and supported a one- or two factor model as demonstrated for pre-school children (Karr et al., 2018). While EF is largely executed by descending neuronal networks originating in the prefrontal cortex in adults (O’Connor et al., 2011; Burgess and Stuss, 2017), young children appear to utilize areas more distributed throughout the brain (Spencer-Smith and Anderson, 2009; Jacobs et al., 2011; Rothbart et al., 2011). Hence, if the integrity of the brain is affected or compromised during childhood, such as by pABI, this may have significant implications for the subsequent development of the neural networks underpinning EF (Stuss and Knight, 2013). In typically developed children, behavior is adapted according to changes in the environment. However, when EF impairments are present, significant delays may be observed in cognitive, social, and academic development (Leventer and De Luca, 2010).

The assessment of EF is challenging. Firstly, there is a lack of consensus about the construct of EF (Jurado and Rosselli, 2007; Chan et al., 2008), leading to a somewhat disagreement about what EF construct a given test measures (Karr et al., 2018). Secondly, tests of EF should typically assess controlled actions, be novel, complex, involve the integration of information, require the ability to form goal-directed plans and change strategies when needed (Lezak et al., 2004). However, the contrast between controlled actions and automatic actions are at the opposite ends of a continuum, and performance on a novel task will over time shift gradually from controlled to automatic (Hughes and Graham, 2002). Also, the requirement of “novelty” makes it difficult for measures of EF to reach good reliability as the task is no longer novel after the first administration (Burgess, 1997; Salthouse et al., 2003). The multidimensional structure of many EF tests requires many different cognitive abilities interacting to enable a successful performance (Hughes and Graham, 2002; Duggan and Garcia-Barrera, 2015). This gives rise to the problem of “task impurity” as the task often also requires the use of non-executive cognitive processes as well as more than one EF process (Burgess, 1997). Finally, the highly structured and examiner-guided setting in which the examination typically takes place, makes less demand on the person’s goal setting, structuring, and decision-making abilities than the real life setting (Stuss and Alexander, 2000).

Assessment of EF in children has further been hampered by the fact that pediatric neuropsychological tests are often adapted from adult versions (Anderson, 2001), rather than primarily developed for children based on a developmental model of EF. In addition, most research has examined EF within narrow age ranges, making comparisons across age groups from infancy to adolescence difficult. Due to the prolonged developmental trajectories of EF (Bunge et al., 2002; Lenroot et al., 2007), it is difficult to develop tasks that are appropriate for different age groups as the tasks for pre-school children are too easy or do not capture EF among adolescents, and the most complex tasks for adolescents are not appropriate for young children as they may target aspects of EF that are yet to develop (Best and Miller, 2010).

The inherent complexity of EF, particularly the aspects of goal setting and plan execution (Burgess and Shallice, 1991; Burgess et al., 1998; Royall et al., 2002), makes it challenging to assess in formalized settings (Manchester et al., 2004). Standard neuropsychological tests that typically assess cognitive impairment (level of body function in ICF), are designed with high reliability and internal validity in mind. Furthermore, they assess more narrow, situational limited processes and may not fully capture all aspects of EF across various contexts (Goldberg and Podell, 2000). Daily life activities are complex and open-ended, and good performance often relies on several lower-level cognitive functions in addition to EF (Burgess, 1997; Burgess et al., 1998). However, assessment of executive deficits evident in everyday life (level of activity/participation in ICF) is often difficult to capture in laboratory settings. In particular, there is a lack of measures evaluating the function at the level of participation following the ICF framework (World Health Organization, 2001). It has been argued that neuropsychological measures of EF need to possess ecological validity, in order to be useful for informing brain injury rehabilitation (Lewis et al., 2011). Ecological validity refers to the degree to which performance on standardized tests corresponds to the actual behavior of interest in natural settings (Barkley, 1991; Franzen and Wilhelm, 1996; Chaytor et al., 2006), and should therefore have characteristics similar to a naturally occurring behavior and to be able to predict everyday function (Franzen and Wilhelm, 1996). However, today few such tools are available for children (Chevignard et al., 2009, 2012).

Previous studies have evaluated ecological validity in terms of: (1) association between performance-based neuropsychological tests; and (2) self- or parent reports about everyday EF such as the Behavior Rating Inventory of Executive Functions (BRIEF; Gioia et al., 2000) and Dysexecutive Questionnaire for Children (Emslie et al., 2003; the veridicality approach; Chaytor et al., 2006). Moderate to weak correlations have been found between performance-based tests and questionnaires (Toplak et al., 2013). However, few studies have included teacher reports. Another approach has been to administer tests that theoretically resemble the cognitive demands in the everyday environment (the verisimilitude approach; Franzen and Wilhelm, 1996; Emslie et al., 2003; Chaytor et al., 2006). Some are standardized “paper and pencil” tasks, that simulate situations close to everyday life, such as the Behavioral Assessment of Dysexecutive Syndrome for Children (BADS-C; Emslie et al., 2003). Others are real-life situation tasks with observation of actual performance in a natural environment, such as the Do-Eat (Josman et al., 2010; Rosenblum et al., 2015), the Weekly Calendar Planning Assessment (Toglia, 2015), or the Children’s Cooking Task (CCT; Poncet et al., 2015a). While performance-based tests are designed to focus mainly on one distinct EF, ecological tests of EF and questionnaires often try to capture a wider range of EFs. This is more similar to daily life EF but also makes comparisons across assessment types more difficult. Furthermore, while one might assume that questionnaires are the most valid measure of daily life EF, there are several factors that might influence the accuracy of the rating. While self-report may provide information on a person’s subjective experience of their function, reduced self-awareness following pABI (Prigatano and Altman, 1990; Hart et al., 2005) and other self-report biases such as social desirability, recall bias or context effects may affect their rating (Van de Mortel, 2008; Demetriou et al., 2015). Parent reports may be influenced by personal and affective factors, family distress and dynamics (Stokes et al., 2011), and their familiarity with their child’s everyday functioning (Norris and Tate, 2000). Teachers observe the child in a more complex situation compared to the home context (e.g., problem-solving on academic tasks, compliance in the classroom, and social interactions), and are able to evaluate the child’s function relative to their peers.

The Children’s Cooking Task (CCT) is a complex test specifically developed to assess EF in a standardized naturalistic open-ended environment (cooking). It is designed to require task setting and evaluation of goal maintenance and involves multi-tasking abilities, in addition to providing minimal external control and structure. The behavioral rating is based on error analysis in accordance with Lezak’s cognitive model where execution errors are thought to reflect dysfunction in planning, goal direction, and task monitoring (Lezak et al., 2004), as well as Schwartz’s model of action disorganization syndrome (Schwartz et al., 1991; Fogel et al., 2020). However, the complex, multi-component nature of the CCT means that in addition to assessing the ability to actively maintain task goals and goal-related information [common EF (Friedman and Miyake, 2017)], it also taps into distinct EF functions such as inhibition, working memory, cognitive flexibility (Diamond, 2006), as well as integrative, metacognitive functions allowing the person to control, coordinate and integrate also non-executive functions (Stuss and Knight, 2013). As such, it is difficult to disentangle the different functions when trying to pinpoint the exact function responsible for poor performance.

The French and English versions have previously demonstrated good psychometric properties (albeit only in three studies, some of them with small samples) regarding internal consistency, test-retest reliability (Chevignard et al., 2010), and ability to discriminate between typically developing children and children with pABI (Chevignard et al., 2010; Krasny-Pacini et al., 2017a; Fogel et al., 2020), children with developmental dyspraxia (DCD; Toussaint-Thorin et al., 2013), and children with neurodevelopmental disorders (Fogel et al., 2020). Regarding convergent validity, results are mixed. Chevignard and colleagues (Chevignard et al., 2010) found low to moderate associations between total errors on the CCT and performance-based test of EF such as the Trail Making Test (TMT), verbal fluency, sorting task, and 20 questions from the Delis-Kaplan Executive Function System (D-KEFS), and weak to moderate associations to questionnaires assessing EF (Chevignard et al., 2009, 2010), in children with pABI (*n* = 25). Other studies found associations between total errors on CCT and EF questionnaires in children with pABI (*n* = 10; Chevignard et al., 2009) and in children with neurodevelopmental disorders (*n* = 41; Fogel et al., 2020).

In sum, advancing the assessment of EF in children and adolescents is warranted. There is also a need to implement tests that are more sensitive to deficits in everyday activities in a natural environment. The CCT has the potential to detect EF impairments in complex and naturalistic settings. Hence, the aim of the present study was to examine the reliability and validity of a translated and adapted Norwegian version of the CCT in a larger sample including children and adolescents with typical development and pABI. The specific aims were to examine the: (1) internal consistency and inter-rater reliability; (2) sensitivity and specificity i.e., the ability to discriminate between typically developed children and children with pABI, as well as age-related differences in task performance; (3) convergent validity by including three assessment levels; standardized performance-based tests of EF, other ecologically sensitive tests of EF, parental and teacher reports of EF in daily life, and measures of overall disability and functioning in everyday life (global function); and (4) divergent validity by comparing CCT-performance to IQ, processing speed, memory, and learning. We hypothesized that we would confirm findings from earlier studies reporting good internal consistency and inter-rater reliability (Chevignard et al., 2009, 2010) and that children with pABI would make more errors than the typically developing children (Chevignard et al., 2009, 2010; Krasny-Pacini et al., 2017a). Although we expected that performance on the CCT would be associated with measures of EF, but not with measures of other cognitive functions, the CCT is a complex task that may trigger a number of cognitive functions in order to complete the task. Furthermore, previous studies have demonstrated associations between CCT and measures of IQ (Chevignard et al., 2010). Hence, the analyses of convergent and divergent validity are of an exploratory nature.

## Material and Methods

### Study Design and Participants

The present study has a cross-sectional design, based on baseline data derived from a multicenter RCT (Hypher et al., 2019) with 75 pABI participants and 59 healthy controls (HCs).

Inclusion criteria included: (i) age 10–17 years; (ii) a diagnosis of pABI; (iii) a period of at least 12 months since injury/illness diagnosis, or 12 months since completion of cancer treatment; and (iv) executive dysfunction in daily life as determined by a semi-structured interview. Exclusion criteria were as follows: (i) injury acquired before 2 years of age; (ii) cognitive, sensory, physical, or language impairment affecting the capacity to attend regular school (i.e., unable to follow educational goals of peers and regular classroom teaching, even with adaptation or extra help); (iii) pre-injury neurological or developmental disease, severe (neuro)psychiatric disorder and/or use of stimulant medication; (iv) recently detected brain tumor relapse; (v) not fluent in Norwegian; and (vi) food allergy interfering with the ability to complete the CCT.

For the healthy control (HC) group, typically developed children aged 10–17 years were included. Exclusion criteria were: (i) previous head injury with loss of consciousness >5 min, stroke, other brain injuries, brain diseases or developmental condition; (ii) severe (neuro)psychiatric disorder or substance abuse; (iii) cognitive, sensory, physical, or language impairment affecting the capacity to attend mainstream school; (iv) not fluent in Norwegian; and (v) food allergy interfering with the ability to complete the CCT.

### Recruitment and Assessment Procedures

Data were collected between 2017 and 2019 at St. Olav’s Hospital, Trondheim University Hospital, and Oslo University Hospital. Participants were recruited from the trauma referral centers for the Central, South-Eastern, and Northern regions of Norway. Seventy-seven participants with pABI who met inclusion and exclusion criteria and completed baseline assessment in the RCT study were included in this study. For a full description of recruitment procedures, see Hypher et al. (2019, 2021) and Brandt et al. (2021). In addition, further two participants with severe motor and language dysfunction but who were able to attend regular school, were unable to complete the CCT without adult interference and were thus excluded. So, data from 75 participants with pABI were available for analysis in the present study.

The healthy control group (HCs, *n* = 59) was recruited from local public schools. Written informed consent was required from potential participants (≥16 years) or primary caregivers (participants <16 years). The HC’s were designed to recruit an equal number of boys and girls, and with an age span evenly distributed across three age groups (10–11 years, 12–13 years, 14–17 years).

### Measures

#### Demographics and Background

Age, sex, level of parental education, cohabitation, and family function were collected from all participants. For the pABI participants, we extracted medical and injury characteristics from medical records and a structured interview. This included results from brain imaging such as computerized tomography (CT) and magnetic resonance imaging (MRI) taken during the first year post injury or disease onset.

#### Estimate of IQ

We employed two measures of IQ from the Wechsler Intelligence Scales for Children, 5th edition (WISC-V; Wechsler, 2014): the Verbal Comprehension Index (VCI, calculated on basis of the Vocabulary and Similarities subtests; Lezak et al., 2004), and a composite score of the Visual Spatial Index (VSI), calculated based on the Block Design and Matrix Reasoning subtests where raw scores were converted to scaled scores, and the sum of scaled scores were divided on the number of tests (*n* = 2). Additionally, the Processing Speed Index (PSI) was derived from the Coding and Symbol Search subtests for children with pABI.

#### Children’s Cooking Task

The CCT was originally developed in French (Chevignard et al., 2009), and translated and adapted to English by Chevignard et al. (2010) and Poncet et al. (2015a). In the CCT, the child is asked to prepare a chocolate cake and a fruit drink by following structured recipes in the form of a checklist with numbered steps and pictures, placed in a structured, child-friendly recipe book (with a few distractor recipes). The child is asked to follow the recipes and instructions scrupulously, even if this somewhat deviates from their personal everyday routine (e.g., not fetching food in the refrigerator as all ingredients are ready on the table, putting the dirty items in the sink rather than in the dishwasher). Standardized ingredients and utensils are set on the table, including distractor items.

The examiner records all the child’s actions and commentaries (written or video) and subsequently rates the errors. The CCT allows for a two-step error rating where descriptive errors are rated first, and a second step where errors are reconsidered in terms of the reason why they occurred (neuropsychological rating). Subclassification of errors on the descriptive level provides an overview of the number of additions, omissions, comments, and questions, substitutions-sequence errors, and estimation errors. The total number of errors is calculated as the total of those five types of errors. Subclassification of errors based on neuropsychological concepts provides an overview of the number of control errors, context neglect, environmental adherence, purposeless actions and displacements, dependency, and inappropriate behavior. In addition, the test provides qualitative measures (goal achievement, occurrence of dangerous behaviors, and need for adult intervention). Norms are not yet available. The total number of errors was used as the primary outcome in analyses of reliability and validity, and the total number of errors without comments or questions was used as a secondary outcome (this allows focusing on the “action errors”, without taking into account the child’s comments or questions).

The English version was translated (double-translation and reconciliation procedure) and adapted into Norwegian according to the ITC Guidelines for Translating and Adapting Tests (International Test Commission, 2017) by the first author (TF) and evaluated and accepted by Dr. MC. We developed a Norwegian manual describing administration, instructions, and scoring criteria, including minor changes to grocery items and kitchen utensils that are more common in Norway. Four healthy children between the age of 11 and 15 years piloted the CCT and gave feedback on the instructions and recipe book, as well as an assessment of how feasible and enjoyable the test was.

Inter-rater reliability for the CCT was assessed for 30 participants (14 children with pABI and 16 HCs) by two independent raters.

#### Measures of Convergent Validity (Executive Function)

All performance-based tests described below have demonstrated adequate validity and reliability, and most have been recommended by McCauley et al. (2012) as common outcome measures after TBI.

Although there is a debate on the structure of EF (Karr et al., 2018), we selected neuropsychological tests assessing the three executive processes described in Diamond’s model of EF; working memory, cognitive flexibility, and inhibition (Diamond, 2006) were used as criterion measures for convergent validity of the CCT. In addition, because of the multicomponent nature of CCT, we chose tests that are assumed to assess planning (including problem-solving). The tests were only administered to participants with pABI. Measures of *EF Inhibition* include time score (raw score) from the Color-Word Interference Test—condition 3 [CWIT3—inhibition, from the Delis-Kaplan Executive Function System (D-KEFS); Delis et al., 2001], as well as the number of commissions and perseverations (*T*-scores) from Conner’s Continuous Performance Test 3rd Edition (CPT-III; Keith Conners et al., 2018). Measures of *EF Cognitive flexibility* include time score (raw score) from CWIT, condition 4 (CWIT4—inhibition/switch), and time score (raw score) from Trail Making Test, condition 4 (TMT4—number-letter switching, from the D-KEFS). Measures of *EF Working memory* include total score (raw score) from the Digit Span and Picture Span from WISC-V. Measures of *EF planning* include the total move score (raw score) from the Tower of London (ToL; Culbertson and Zillmer, 2006). For CWIT and TMT a high raw score indicates poor performance. However, a higher T-score on the C-CPT as well as a high raw score on the Digit Span and Picture Span indicate better performance. In addition, we calculated composite scores as the mean standard test score for each domain.

To assess EF in everyday life for the pABI group, we applied the total age and gender standardized score from the three composite indices: Global Executive Composite (GEC), Behavior Regulation Index (BRI), and Metacognitive Index (MI) from the BRIEF (Gioia et al., 2000) utilizing self-report, parent report, and teacher report. Higher *T*-scores indicate worse executive functioning in everyday life.

Global outcome (i.e., ability to resume independent living, education, and leisure activities) was assessed by the Glasgow Outcome Scale Extended—pediatric version (GOSE peds; Beers et al., 2012) for participants with pABI. The scale rates global outcome from 1 (death), 2 (vegetative state), 3–6 as severe/moderate disability, and 7 as lower good recovery, and 8 as upper good recovery.

BADS-C was administered to both the pABI group and the control group. The BADS-C is considered to be an ecological test of EF and has previously been used as a criterion measure of convergent and ecological validity for the CCT (Emslie et al., 2003). In BADS-C, different aspects of EF are examined by six performance-based subtests (Playing Cards Test; Water Test; Zoo Map Tests 1 and 2; Key Search Test; and the Six Parts Test), and a questionnaire (DEX-C). The battery measures shifting, planning and goal-directed behavior, estimation abilities, and response inhibition. Raw scores are converted to scaled scores and provide a total score (Emslie et al., 2003). As raw scores on the subtests from the BADS-C may result in negative numbers, we used age-adjusted scaled scores in the analyses. High-scaled scores indicate better performance. Furthermore, we used the total score (raw score) from the DEX-C from BADS-C, utilizing the parent report and teacher report for participants with pABI, and parent report from HCs. A high score on DEX-C indicates executive difficulties.

#### Measures of Divergent Validity

Although recognizing that the complex nature of the CCT may implicate integration of non-executive functions, and that measures of cognitive functions such as working memory, attention, and learning may require the integration of all these functions, we chose nevertheless to categorize the following measures as assessing non-executive function (divergent validity): Processing speed (total raw score) from the Coding and Symbol Search subtests from WISC-V, time raw score from condition 1 and 2 from CWIT, and time raw score from condition 2 from TMT, total Learning (sum of recalled words in trial 3–5), immediate and delayed recall from the Children’s Auditory Verbal Learning Test 2nd (CAVLT-2; Talley, 1993) and Attention [number of omissions, detection (*d*), Hit Reaction Time Standard Deviation and Hit reaction time block change from the C-CPT-III (*T*-scores)]. For raw scores on Coding, Symbol Search, and CWIT a higher score indicates poorer function. For raw scores on CAVLT and *T*-scores on C-CPT-III, a higher score indicates better function.

#### Assessment Procedure

Participants in the pABI group were assessed at the hospital during the course of one workday by experienced test-technicians and psychology students (master level). The day was divided into four blocks (Block 1 included WISC-V, Block 2 included the remaining neuropsychological tests, Block 3 included CCT and BADS-C, and Block 4 included questionnaires). Participants were randomly allocated to each of the blocks for assessment. As such, the order of test administration varied between the participants. The assessment was limited to 1 day to prevent unnecessary use of the participants’ time and the burden of additional travel, as we recruited from the whole country with long travel distances. To alleviate tiredness, participants were given frequent breaks and a one-hour lunch break. To compensate for variation associated with multiple assessors, a Standard Operating Procedure (SOP) described the protocol and procedures for assessment, and the test administrators received training from an experienced clinical neuropsychologist. Participants, their parents, and teachers completed the BRIEF (Gioia et al., 2000), and parents and teachers completed the DEX-C (Emslie et al., 2003).

The HCs were assessed by the same test administrator at each site. However, the HCs were only assessed with the WISC-V subscales Vocabulary, Similarities, Block Design, Matrix Reasoning (Brandt et al., 2021); the CCT, and the BADS-C (including parent report from the DEX-C). The order of test presentation varied across participants.

Secure and anonymous management of the data across both sites was ensured by the use of a Web Clinical Research Form (webCRF).

### Ethics

Study procedures and monitoring were performed according to ICH Guideline for Good Clinical Practice and Norwegian procedures and regulations for Clinical Trials, described by the Norwegian Clinical Studies Infrastructure Network. Written informed consent was signed by participants (>16 years) or primary caregivers (<16 years). The study was approved by the Regional Committees for Medical and Health Research Ethics, Norway (2017/772/REK), and conducted in accordance with principles of the Helsinki Declaration and the standards for Ethical Research Involving Children (ChildWatch International and UNICEF). Clinical Trial Registration No.: NCT03215342.

### Statistical Analysis

Dependent variables were checked for normality by use of the Shapiro-Wilks test and inspection of Q-Q plots. Demographic and injury severity characteristics and cognitive sub-tests are presented as mean (± *standard deviation, SD*) for normally distributed data, and otherwise as median with interquartile range (IQR). All data analyses were conducted using IBM-SPSS version 27 (IBM Corp, 2020).

In the presence of missing data, we used available case analysis, utilizing all cases for which a variable is present. Thus, the number of cases differs for each variable. Reported *p*-values are two-sided. Due to multiple assessments, a pragmatic α-level of ≤0.01 was considered significant, chosen to partially account for multiple testing. A more formal approach to control for multiple comparisons (e.g., Bonferroni corrections) was considered too conservative in the current study given its exploratory nature (Perneger, 1998). To avoid an inflated Type 2 error, we chose to consider an α-level between 0.05 and 0.01 as a trend toward significance.

For non-normally distributed data, nonparametric statistics were employed. Proportions were compared using the chi-square test, the unconditional *z*-pooled test (Altman et al., 2001), and the Newcombe confidence interval (Lydersen et al., 2012; Fagerland et al., 2015). Between group differences were investigated by Mann–Whitney *U*-test and Kruskal-Wallis test (continuous variables). As data were non-normally distributed, we evaluated the sensitivity and specificity of the different error types on CCT by calculating a Receiver Operator Characteristic (ROC) Curve and calculated the area under the ROC (AUROC). AUROC between 0.9 and 1.0 were considered Very good, 0.8–0.9 were considered Good, 0.7–0.8 were considered Fair, 0.6–0.7 were considered Poor, and 0.5–0.6 would be considered as Fail.

For normally distributed data, parametric statistics were employed. Comparisons of groups with regard to age at testing and estimated verbal IQ and visual IQ composite score were evaluated by independent samples *t*-test. To explore the clinical significance of the differences between groups, the effect size was calculated (*r*) for comparisons using Mann-Whitney *U*-test, and Glass’ delta (*d*) for comparisons using *t*-tests (Field, 2013). Effect size (ES) is reported as *r* defining small ES as *r* = 0.1–0.3; medium ES as *r* = 0.3–0.5; large ES as *r* > 0.5 (Field, 2013).

Internal consistency was calculated for total errors on CCT by Cronbach’s alpha (α), by first entering all error types from the CCT in the analysis, and then observing the stability of the measure by removing each individual error type from the model. Inter-rater reliability for total errors on the CCT was assessed by using single-rating (*k* = 2), consistency-agreement, and a two-way random-effects model for the intraclass correlation (ICC; Field, 2005). Values of ICC were considered excellent (>0.90), good (0.75–0.90), fair (0.50–0.75), and poor (<0.50; Perinetti, 2018).

To study convergent and divergent validity, Spearman rank order correlation (rho) was used to estimate the association between the primary and secondary outcome measures in CCT and performance-based tests and questionnaires.

## Results

### Description of Participants

Demographic and background characteristics are presented in Table 1. The HCs and pABI groups did not differ significantly in terms of distribution of sex, age at assessment, or number of participants living in intact family units. However, parental education was significantly higher in the HCs. Also, the HC had higher intellectual abilities than the pABI group both in terms of verbal comprehension IQ (*t*_(128)_ = −5.75, *p* < 0.001, Glass’ *d*: −1.0) and visual IQ composite score (*t*_(131)_ = −5.23, *p* < 0.001, Glass’ *d*: −0.99).

Comparing the pABI group and the HCs on selected measures of EF examined for both groups, we found that HC had better performance on BADS-C overall scaled score (*t*_(131.8)_ = 5.871, *p* < 0.001, Glass’ *d*: 0.92) and their parents reported less EF problems on the DEX-C (*t*_(130.1)_ = −7.576, *p* < 0.001, Glass’ *d*: −1.13). Mean and SD is given in the tables below.

**Table 1:** Description of participants: demographics, time since injury, injury characteristics, IQ, and clinical observations at the time of testing.

Variable demographics	*n*	Children with pABI	*n*	Healthy controls	*p*-value
Male sex (*n*, %)	75	33 (44)	59	29 (49)	0.487*
Age at assessment (*M*, *SD*)	75	13.4 (2.3)	59	12.8 (1.9)	0.083^†^
Age at injury (*M*, *SD*/range)	75	8.0 (3.6/1–15)			
Maternal education (*n*, %)	71		59		<0.001*
Complete secondary school		4 (5.6)		0
Complete gymnasium		23 (32.4)		2 (3.3)	
Attended college/university		44 (62.0)		57 (96.7)
Paternal education (*n*, %)	66		58		<0.001*
Complete secondary school		5 (7.6)		2 (3.4)	
Complete gymnasium		31 (47)		8 (14)	
Attended college/university		30 (55)		48 (83)	
Intact family unit (*n*, %)	75	48 (64)	58	43 (74)	0.408*
**Type of injury/disease (*n*, %)**	75	
TBI		21 (28.0)	
Brain tumor		27 (36.0)	
Cerebrovascular insult		15 (20.0)	
Hypoxia/anoxia		5 (6.7)	
Brain infection/inflammation		7 (9.3)	
**Imaging (*n*, %)**	75		
Confirmatory findings on brain imaging scans	67		88	
Estimated IQ at time of assessment (*M*, *SD*)				
Verbal comprehension index (VCI), IQ scores	73	96.6 (11.9)	57	108.8 (12.3)	<0.001^†^
Visual IQ composite score (scaled score)	74	9.1 (2.4)	59	11.2 (2.1)	<0.001^†^
**Global outcome at assessment** (GOS-E peds score, *n*, %)	70
GOS-E peds score 3 (Lower Severe Disability)		8 (11.4)			
GOS-E peds score 4 (Upper severe Disability)		6 (8.6)	
GOS-E peds score 5 (Lower Moderate Disability)		4 (5.7)	
GOS-E peds score 6(Upper Moderate Disability)		34 (48.6)	
GOS-E peds score 7 Lower Good Recovery)		12 (17.1)	
GOS-E peds score 8 Upper Good Recovery		6 (8.6)	

pABI, Pediatric Acquired Brain Injury; GOS-E peds, Pediatric Glasgow Outcome Scale Extended; IQR, inter quartile range; SD, standard deviation; TAI, traumatic diffuse axonal injury; TBI, traumatic brain injury. *Pearson’s Chi-squared test. ^†^Independent samples *t*-test.

### Measures of Reliability

Internal consistency of the CCT was acceptable (*α* = 0.85) when entering all scores for the different types of error in the analysis. Internal consistency was relatively stable between α = 0.82 and 0.86 when each individual error type was removed from the model.

Good inter-rater reliability was found for total errors on the CCT using single-rating (*k* = 2), consistency-agreement, two-way random-effects model for the intraclass correlation (ICC, estimate 0.865, 95% CI = 0.735–0.933, *F* = 13.764, *df* = 29, *p* = < 0.001). Inter-rater reliability estimate varied between 0.529 (estimation errors) and 0.931 (context neglect errors) for the different error scores (Table 2).

**Table 2:** Intraclass correlation (ICC) for different error types of Children’s Cooking Task (CCT) using single-rating, consistency-agreement, two-way random-effects model.

Single measures from the CCT	Intraclass correlation (ICC)	95% Confidence Interval (CI)		*F*-test with true value 0			
*N* = 30		Lower bound	Upper bound	Value	*df*1	*df*2	*p*-value
Total errors	0.865	0.735	0.933	13.764	29	29	<0.001
Total errors without comments and questions	0.787	0.600	0.893	8.401	29	29	<0.001
Additions	0.696	0.453	0.843	5.579	29	29	<0.001
Omissions	0.902	0.805	0.952	19.477	29	29	<0.001
Comments and questions	0.856	0.720	0.929	12.918	29	29	<0.001
Substitution-Sequence errors	0.610	0.325	0.793	4.128	29	29	<0.001
Estimation errors	0.529	0.214	0.744	3.243	29	29	0.001
Control errors	0.807	0.633	0.903	9.337	29	29	<0.001
Context neglect errors	0.931	0.861	0.967	28.092	29	29	<0.001
Environmental adherence errors	0.686	0.438	0.837	5.375	29	29	<0.001
Purposeless actions and displacements	0.625	0.346	0.802	4.326	29	29	<0.001
Dependency	0.844	0.697	0.922	11.782	29	29	<0.001
Inappropriate behavior	0.624	0.345	0.801	4.314	29	29	<0.001
Prospective memory total	0.715	0.483	0.854	6.028	29	29	<0.001

### Performance on the CCT and Evaluation of Sensitivity and Specificity

For the qualitative scores, a larger proportion of children with pABI failed to achieve the overall goal of the task (*n* = 10, 13.3%) compared to the HC (*n* = 1, 1.7%, the difference between proportions: 12%; Newcombe CI (95%): 2%–21%; the exact unconditional z-pooled test: *p* = 0.05). In addition, a larger proportion of children with pABI needed intervention from an adult to complete the task (*n* = 19, 25.3%) compared to the HC (*n* = 1, 1.7%, difference between proportions: 24%; CI: 12%-35%, *p* = 0.05). There was no difference between the groups with regard to the occurrence of dangerous behavior (pABI: *n* = 9 vs. HC: *n* = 12).

**Figure 1:**
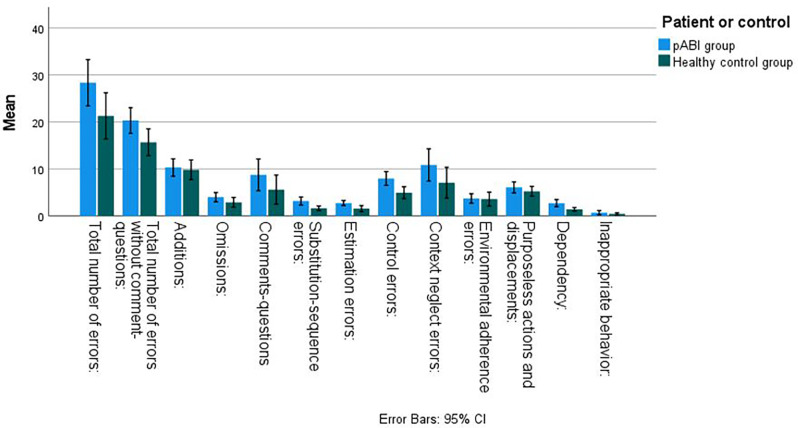
Number of errors on the Childrens Cooking Task for children with pABI and HCs.

Performance on the quantitative measures of CCT for the pABI group and the HCs is presented in Table 3, Figure 1. The pABI group made significantly more errors in total on the CCT compared to the HCs, even when removing the number of comments and questions (considering only the action errors). In addition, the pABI group made significantly more of the following types of errors: estimation errors, control errors, context neglect, and dependency. Relative to adjusted alpha, there was a trend toward significant difference between groups in the number of comments and questions, and substitutions-sequence errors. Effect sizes were in the moderate to small range.

**Table 3:** Comparison of the number of errors on the Children’s Cooking Task in the pABI group and HCs.

Item	pABI (*n* = 75)		HC (*n* = 59)		Mann-Whitney	*p*-value	(*z*)	Effect size
	Median	IQR (range)	Median	IQR (range)	*U* test			(*r*)
Total errors	23	21 (4–115)	18	12 (4–132)	1,636.5	0.01	−2.583	−0.22
Total errors without comments and questions	18	15 (4–59)	13	9 (2–58)	1,603.0	0.006	−2.735	−0.24
*Descriptive level*								
Additions	8	8 (0–40)	8	6 (1–42)	2,175.0	0.866	−0.169	−0.01
Omissions	3	5 (0–18)	1	2 (0–17)	1,804.5	0.063	−1.858	−0.16
Commentaries and questions	4	8 (0–75)	2	4 (0–74)	1,729.0	0.029	−2.182	−0.19
Substitutions-Sequence errors	2	4 (0–18)	1	2 (0–9)	1,696.0	0.018	−2.365	−0.20
Estimation errors	2	3 (0–10)	1	2 (0–18)	1,273.0	<0.001	−4.303	−0.37
*Neuropsychological classification*							
Control errors	7	8 (1–30)	4	5 (0–23)	1,479.0	0.001	−3.302	−0.29
Context neglect	7	8 (0–77)	4	5 (1–82)	1,638.5	0.01	−2.583	−0.22
Environmental adherence	3	4 (0–26)	3	3 (0–40)	2,094.5	0.593	−0.534	−0.05
Purposeless actions and displacements	5	6 (0–25)	5	5 (0–23)	2,079.5	0.549	−0.599	−0.05
Dependency	2	3 (0–23)	1	2 (0–7)	1,599.0	0.005	−2.813	−0.24
Inappropriate behavior	0	1 (0–14)	0	1 (0–4)	2,150.5	0.736	−0.337	−0.03

*pABI, pediatric Acquired Brain Injury; HCs, Healthy Controls; IQR, Inter-quartile range*.

**Figure 2:**
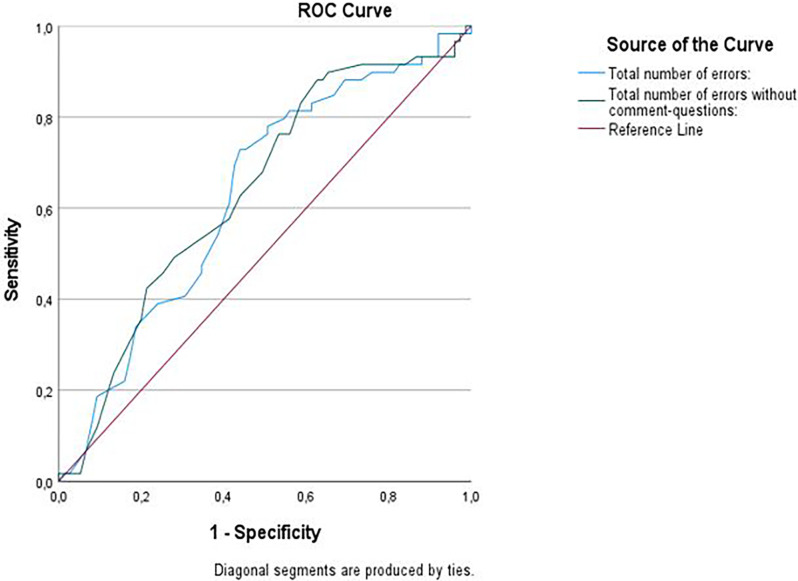
ROC Curve for total errors and total errors without comments and questions on Childrens Cooking Task (CCT).

When evaluating the sensitivity and specificity of the quantitative measures of CCT, we calculated ROC for all error types (Table 4, Figure 2). Both total errors on CCT and total errors when removing the number of comments and questions considering only the action errors significantly discriminated between the groups. However, the ROC calculations were classified as Poor. The error type Estimation errors were considered best at discriminating between the groups and were classified as Fair. When applying adjusted alpha, we also found that control errors, context neglect errors, and dependency errors significantly discriminated between the groups. However, these were also classified as Poor.

**Table 4:** ROC analysis of sensitivity and specificity of the different error types on the Children’s Cooking Task.

CCT error type	AUROC	SE	*p*-value	95% CI		Classification
				Lower	Upper	
Total errors	0.630	0.048	0.010	0.535	0.725	Poor
Total errors without questions and comments	0.638	0.048	0.006	0.543	0.732	Poor
Additions	0.508	0.050	0.867	0.410	0.607	Fail
Omissions	0.592	0.050	0.067	0.495	0.690	Fail
Comments-questions	0.609	0.049	0.030	0.513	0.705	Poor
Substitution-sequence errors	0.617	0.048	0.021	0.522	0.711	Poor
Estimation errors	0.712	0.045	<0.001	0.624	0.801	Fair
Control errors	0.666	0.047	0.001	0.574	0.758	Poor
Context neglect errors	0.630	0.048	0.010	0.535	0.724	Poor
Environmental adherence errors	0.527	0.05	0.597	0.428	0.625	Fail
Purposeless actions and displacements	0.530	0.050	0.551	0.432	0.628	Fail
Dependency	0.639	0.047	0.006	0.546	0.732	Poor
Inappropriate behavior	0.514	0.050	0.781	0.415	0.613	Fail

*Abbreviations: ROC, receiver operating curve; AUROC, area under the receiver operating curve; SE, standard error; CI, confidence interval*.

### The Effect of Age, Parental Education, and Functional Outcome on CCT

Exploring the effect of age on total errors on CCT, we observed that lower age at assessment was associated with more errors on the CCT in the pABI group (*n* = 75, rho: −0.369, *p* = 0.001). We found that younger children with pABI made more additions (*n* = 75, rho: −0.353, *p* = 0.002), omissions (*n* = 75, rho: −0.302, *p* = 0.008), estimation errors (*n* = 75, rho: −0.395, *p* < 0.001), and had more inappropriate behavior (*n* = 75, rho: −0.339, *p* = 0.003).

In the HC group, there was no significant association between the age at assessment and the total number of errors on CCT, although younger age at assessment was associated with a higher number of omissions (*n* = 59, rho: −0.408, *p* = 0.001).

Neither maternal nor paternal education was associated with total errors on CCT for any of the groups (data not shown).

A higher number of total errors on CCT was associated with lower functional outcomes (pGOSE; rho: −0.314, *p* = 0.007) among children with pABI.

### Convergent Validity—Association Between CCT and Other Measures of EF

Tests of convergent validity in the pABI group are summarized in Table 5.

**Table 5:** Convergent validity—associations between the total number of errors on Children’s Cooking Task (CCT) and other measures of executive function (EF) in children with pABI.

		Performance on test	CCT total errors	
Type of measure	*n*	Mean (SD)	Spearman’s rho	*p*-value
Domain score Executive Function (EF)*	71		0.025	0.833
EF Inhibition**	74		0.188	0.109
D-KEFS CWIT3: Inhibition time, raw score	75	76.7 (25.8)	0.335	0.003
CPT-III: Commissions, *T*-score	74	53.3 (7.9)	0.258	0.027
CPT-III: Perseverations, *T*-score	74	60.3 (14.7)	0.147	0.210
EF Cognitive flexibility***	75		-0.280	0.015
D-KEFS CWIT4: Inhib/switch, raw score	75	83.3 (26.9)	0.421	<0.001
D-KEFS TMT 4: Subtest 4 time, raw score	75	104.3 (53.4)	0.403	<0.001
EF Planning****	73		-0.014	0.908
Tower of London: Move raw score	73	32.3 (14.1)	0.035	0.763
EF Working memory*****	72		-0.244	0.039
WISC-V Digit span total, raw score	72	25.5 (4.7)	-0.255	0.030
WISC-V Picture memory, raw score	72	28.9 (7.2)	-0.372	0.001
*EF Ecologically sensitive test (BADS-C)*				
Overall scaled score	75	53.5 (10.0)	-0.375	<0.001
Playing Cards Test, raw score	75	1.2 (1.7)	0.262	0.023
Water Test, raw score	75	8.6 (2.0)	-0.259	0.025
Key Search Test, raw score	75	9.8 (4.7)	-0.436	<0.001
Zoo Map Test 1, raw score	75	2.0 (5.7)	-0.308	0.007
Zoo Map Test 2, raw score	75	7.2 (1.6)	0.025	0.831
Six Part Test, raw score	75	11.0 (3.8)	-0.240	0.038
*EF Behavioral regulation*		
DEX-C Parent report, total sum score	75	26.9 (12.7)	-0.019	0.873
DEX-C Teacher report, sum score	70	19.0 (14.6)	0.410	<0.001
BRIEF Self report: GEC, sum score	74	134.6 (28.3)	0.092	0.433
BRIEF Parent report: GEC, sum score	74	133.3 (26.9)	0.128	0.275
BRIEF Teacher report: GEC, sum score	70	108.8 (31.4)	0.374	0.001

Abbreviations: pABI, Pediatric Acquired Brain Injury; D-KEFS, Delis-Kaplan Executive Function System; CWIT, Color-Word Inhibition Test; TMT, Trail Making Test; CPT-III, Conners’s Continuous Performance Test 3rd edition; WISC-V, Wechsler Intelligence Scales for Children 5th edition; BADS-C, Behavioral Assessment of the Dysexecutive Syndrome in Children, DEX-C, Dysexecutive Questionnaire for Children; BRIEF, Behavior Rating Inventory of Executive Function; GEC, Global Executive Composite. *Domain score Executive Function consists of: CWIT 3: Inhibition time scaled score, CWIT 4: Inhibition/switch time scaled score, TMT 4, time scaled score, CPT-III Commissions T-score, CPT-III Perseverations T-score, Tower of London total move T-score, WISC-V Digit Span total scaled score, WISC-V Picture Memory total scaled score. **Domain score EF Inhibition consists of: CWIT 3: Inhibition time, CPT-III Commissions and Perseverations. ***Domain score EF Switching consists of: CWIT 4: Inhibition/switch, TMT 4. ****Domain score EF Planning consists of: Tower of London: Move *T*-score. *****Domain score EF Updating/Working Memory consists of WISC-V Digit span total scaled score, WISC-V Picture memory total scaled score.

Converting the raw scores in Table 5 to scaled scores by means of age-adjusted norms gave the following results: From the Domain EF Inhibition (CWIT3: mean scaled score: 8.1, SD: 3.3); the Domain Cognitive flexibility (CWIT4: mean scaled score: 8.0; SD: 3.1; TMT4 mean scaled score: 8.2, SD: 3.6); Domain EF Planning (ToL standard score: 94.3, SD: 16.5) and Domain Working Memory (WISC Digit span: mean scaled score: 9.0, SD: 2.8; WISC-V Picture memory: mean scaled score: 8.9, SD: 3.0). For the BRIEF: Self-reported GEC: mean T-score: 54.0, SD: 12.9; Parent-reported GEC: 60.2, SD: 11.0; Teacher-reported GEC: 61.1, SD: 16.1.

Fewer total errors on the CCT in children with pABI were associated with better performance on some of the performance-based tests assessing Inhibition (CWIT3 inhibition, time score), Cognitive flexibility (the composite score of Cognitive flexibility, CWIT4 time score, and TMT4 time score), and the Working memory domain (Picture memory) when applying adjusted alpha. There was a trend toward a significant association between the total number of errors on CCT and the CPT-III Commissions from the Inhibition domain, the composite score of Working memory, and Digit span from the Working memory domain. Performance on CCT was not associated with any of the tests assessing Planning (ToL Total Move Score) nor with other EF subtests. Effect sizes were in the moderate to small range.

Furthermore, fewer total errors on the CCT were associated with better overall performance on the ecologically sensitive BADS-C test. Among the BADS-C battery, worse performance on some subtests was also significantly associated with more errors on the CCT (Key Search Test and Zoo Map Test 1, and a trend toward association with poorer performance on Playing Card Test, Water Test, and Six Part Test). Effect sizes were in the moderate to small range.

Notably, fewer total errors with or without comments and questions on the CCT were associated with better perceived daily life EF (DEX-C and BRIEF)—but only through teacher reports, with effect sizes in the moderate range. No associations were observed for self- or parent-report.

Lastly, we investigated the association between CCT and level of disability and functional outcomes. We found that better performance on the CCT was associated with less disability and better functional outcome (GOS-E Peds: *r_s_* = 0.314, *p* = 0.007).

Test of convergent validity in the HC group are summarized in Table 6. Fewer total errors CCT was associated with better overall performance on the BADS-C. On the subtest level, we found more errors on CCT to be associated with poorer performance on the Six Part Test and a trend toward significant association with performance on the Playing Card Test. Effect sizes were in the moderate range. There was no association between CCT and parent report on the DEX-C.

**Table 6:** Convergent validity—association between the total number of errors on the Children’s Cooking Task (CCT) and other measures of executive function—BADS-C and DEX-C in HCs.

		Performance on test	CCT total errors	
Type of measure	*n*	Mean (SD)	Spearman’s rho	*p*-value
*EF Ecologically sensitive test (BADS-C)*	59			
Overall scaled score		102.6 (15.8)	−0.396	0.002
Playing Cards Test raw score		0.7 (0.9)	0.308	0.018
Water Test raw score		9.4 (1.5)	−0.109	0.413
Key Search Test raw score		11.9 (3.0)	−0.126	0.341
Zoo Map Test 1 raw score		4.0 (4.3)	−0.260	0.047
Zoo Map Test 2 raw score		7.8 (0.5)	−0.113	0.394
Six Part Test raw score		13.2 (2.4)	−0.359	0.005
*EF Behavioral regulation*				
DEX-C Parent report, total score	58	12.67 (9.0)	0.147	0.269

*Abbreviations: CCT, Children’s Cooking Task; EF, Executive Function; BADS-C, Behavioral Assessment of the Dysexecutive Syndrome in Children; DEX-C, Dysexecutive Questionnaire for Children; SD, Standard deviation*.

### Divergent Validity—Association Between CCT and non-EF Cognitive Functions

Tests of divergent validity in the pABI group is summarized in Table 7. For the pABI group, we found no significant association between total errors on CCT and the Verbal Comprehension IQ score (VCI: *n* = 73, rho = 0.025; *p* = 0.833). However, a moderate association was found between total errors on the CCT and the Visual IQ Composite score (VSI: *n* = 74, rho = −0.373; *p* = 0.001). For the HC group, we found no association neither between total errors on CCT and the VCI score (*n* = 58, rho = −0.144, *p* = 0.280), nor the VSI (*n* = 59, rho = −0.227; *p* = 0.083).

**Table 7:** Divergent validity—association between the total number of errors on Children’s Cooking Task (CCT) and measures of cognitive function within other domains—processing speed, attention, and memory and learning in the pABI group.

		**Performance on test**	**CCT total errors**	
Type of measure	*n*	Mean (SD)	Spearman’s rho	*p*-value
Domain Processing Speed*	72		-0.339	0.004
WISC-V Coding, raw score	72	47.5 (16.6)	-0.500	<0.001
WISC-V Symbol Search, raw score	72	27.0 (7.8)	-0.506	<0.001
D-KEFS CWIT1: Color Naming time	75	39.9 (10.3)	0.380	0.001
D-KEFS CWIT2: Reading time	75	29.9 (8.3)	0.290	0.012
D-KEFS TMT 2: Number sequencing time	75	46.6 (28.3)	0.475	<0.001
D-KEFS TMT 3: Letter sequencing time	75	47.3 (31.1)	0.461	<0.001
Domain Attention**	74		0.167	0.154
CPT-III: Omissions T-score	74	56.6 (13.8)	0.185	0.114
CPT-III: Detection (d) T-score	74	56.3 (8.7)	0.250	0.032
CPT-III: Hit Reaction Time SD T-score	74	60.5 (14.3)	0.245	0.035
CPT-III: Hit Reaction Time Block Change T score	74	53.6 (8.8)	-0.106	0.367
Domain Learning and Memory***	71		-0.116	0.335
CAVLT-2: Learning, SS-score	71	94.7 (20.0)	-0.160	0.182
CAVLT-2: Immediate recall, SS-score	71	93.1 (20.4)	-0.060	0.621
CAVLT 2: Delayed recall, SS-score	71	96.4 (20.0)	-0.131	0.278

Abbreviations: WISC-V, Wechsler Intelligence Scales for Children 5th edition; D-KEFS, Delis-Kaplan Executive Function System; CWIT, Color-Word Inhibition Test; TMT, Trail Making Test; CPT-III, Conners’s Continuous Performance Test 3rd edition; CAVLT-2, Children’s Auditory Verbal Learning Test 2nd edition; SD, Standard Deviation; CCT, Children’s Cooking Task. *Domain score Processing Speed consists of: CWIT 1: Color naming scaled score, CWIT 2: Reading scaled score, TMT 2 number sequencing scaled score, TMT 3 letter sequencing scaled score, WISC-V Coding scaled score, WISC-V Symbol search scaled score. **Domain score Attention consists of: CPT-III Omissions T-score, CPT-III Detection T-score, CPT-III Hit Reaction Time Standard Deviation T-score, CPT-III Hit Reaction Time Block Change T-score. ***Domain score Learning and Memory consists of: CAVLT-2 Learning T-score, CAVLT-2 Immediate recall *T*-score, CAVLT-2 Delayed Recall *T*-score.

Converting the raw scores in Table 6 to scaled scores by means of age-adjusted norms for the pABI group gives the following results: the Domain Processing Speed (WISC Coding: mean scaled score: 8.2, SD: 3.1; WISC Symbol search scaled: mean score: 8.2; SD: 3.1; CWIT1: mean scaled score: 7.6, SD: 3.5; CWIT2: mean scaled score: 8.1, SD: 3.3; TMT2: mean scaled score: 8.6, SD: 3.7; TMT3: mean scaled score: 8.5, SD: 3.9).

For the pABI group, we found that better performance on the CCT was associated with better processing speed—both on the composite score and most of the individual tests. However, there was no significant association between performance on the CCT and performance on tests in the Domain Attention or the Domain Learning and Memory. Nevertheless, there was a trend toward an association between total errors on CCT and performance on CWIT2, CPT-III Detection t score, and CPT-III Hit Reaction Time SD t score, but the effect sizes were small.

## Discussion

The main aim of this study was to evaluate the psychometric properties of the Norwegian adaptation of CCT. Overall, our findings provide evidence for good internal consistency, inter-rater reliability, as well as discriminant, convergent and divergent validity.

### Reliability

Our study demonstrated good internal consistency for the different error types of the CCT, which is in line with findings in previous studies on children with pABI (Chevignard et al., 2009, 2010) and adults with ABI (Chevignard et al., 2008; Poncet et al., 2015b). Expanding current knowledge, we found good inter-rater reliability for the Norwegian version of CCT. Good inter-rater reliability has also been observed in the adult version of CCT—the adult Cooking Task (CT; Chevignard et al., 2008; Poncet et al., 2015b). The best agreement was found for omissions, context neglect, comments/questions, and dependency. Somewhat in contrast, for adults the best agreement was found for addition, adherence, and comments/questions, while context neglect demonstrated low inter-rater agreement (Poncet et al., 2015b). In both the adult and child versions of the cooking task, context neglect pertains to poor assessment of the environment, failure to respect the instructions, or the frame defined for the task. In the adult version, context neglect includes failure to wash hands, search for utensils, or ingredients in closets rather than those laid out on the working space. In the child version context, neglect include behavior such as licking one’s fingers while cooking and playing football in the kitchen. As the children’s behavior rated as context neglect errors more clearly violate norms and expectations in social interactions; this may improve rating accuracy for the individual raters. Further improvement in rating accuracy may also be due to the fact that the authors of the CT and the CCT have recently revised and improved the scoring criteria (Poncet et al., 2015a), and developed a training manual that explicitly describes a number of situations and instructions for rating—all of which were available for the raters in our study.

### Performance on CCT and Evaluation of Sensitivity and Specificity

Children and adolescents with pABI made more errors than typically developing children, which is in line with our hypothesis and previous studies on children with pABI (Chevignard et al., 2009, 2010; Krasny-Pacini et al., 2017b), and children with more heterogeneous etiologies (Fogel et al., 2020). Particularly, the high error rate in the pABI group revealed a reduced ability to monitor their progress in order to act efficiently on goal-directed actions as well as interact efficiently with their environment (i.e., context neglect). This corroborates the findings from previous studies (Chevignard et al., 2009, 2010; Fogel et al., 2020). Furthermore, the pABI participants were less accurate in their actions (estimation errors) and depended more on external aid (i.e., dependency), suggesting a need for backup strategies to enable the child to find appropriate solutions when a problem appears, and difficulties with analyzing and structuring the situation or environment so that it becomes more manageable.

However, we did not find differences between the two groups with regard to other error types. This is in contrast to previous research where significant differences have been seen for all types of errors (Chevignard et al., 2009, 2010; Fogel et al., 2020). In our study, the participants were recruited based on medical records and screened for executive dysfunction in a phone interview. Chevignard et al. (2010) recruited their participants from a rehabilitation department where they had been referred for in- or out-patient rehabilitation following severe pABI responsible for cognitive and behavioral impairments. The participants in the study by Fogel et al. (2020) were classified as having executive dysfunction on the basis of having a *T*-score >65 on the BRIEF (above the clinical cut-off) prior to inclusion. Although the average parent reported GEC on the BRIEF in the pABI group in our study was significantly worse than the normative average (*T*-score of 60), they reported fewer executive problems than in the study by Fogel et al. (2020). Furthermore, the HC group in our study had more variance and was more heterogeneous than the HC group included in the studies by Chevignard et al. (2010) and Fogel et al. (2020). This is supported by the moderate to small effect sizes between the two groups on the CCT in our study. This is interesting in light of the higher level of verbal IQ and higher parental education in the HC group compared to the children in the pABI group. This may suggest that the CCT measures cognitive abilities beyond IQ and socioeconomic background. Furthermore, we had a larger HC group compared to previous studies and used an outreaching recruitment strategy where the children were recruited in a school setting, which made comparisons across studies difficult.

Although children with pABI performed worse than the HC on CCT, we found that the CCT had overall poor ability to identify group classification based on error scores. This contrasts with the study by Fogel et al. (2020) where they found that all error types contributed considerably to group classification. In our study, estimation errors had the best ability to discriminate between groups which corroborates the findings from Fogel et al. (2020). Comparison across these studies may, however, be difficult as they used Fisher’s linear discriminant function analyses to evaluate the sensitivity and specificity (Fogel et al., 2020). This method is based on the underlying assumption that data is normally distributed and is sensitive to outliers, assumptions that could not be applied to our data. The AUROC has the advantage of being averaged across all possible clinical thresholds (Park et al., 2004), and allows for comparison of diagnostic ability across tests. However, it treats sensitivity and specificity as equally important (Halligan et al., 2015)—which may not be the case in a clinical setting. For example—clinicians who want to avoid depriving patients of important treatment might wish for a more sensitive test at the cost of less specificity. Furthermore, the AUROC depends on scores being accurate, reliable, and not too open for interpretation (Halligan et al., 2015). This means that rating types of behavior (errors) that are more clearly inappropriate within that setting, or developing more stringent criteria for scoring may have an impact on the classification of the discriminative ability of the test in the future (Halligan et al., 2015). Our study indicates that estimation errors may to some extent differentiate between groups. However, our results also suggest that further evaluation of the test’s sensitivity and specificity in future studies with a larger sample size is warranted.

### The Effect of Age on CCT

Expanding current knowledge, we detected a significant age-effect on the CCT among the pABI group. In the pABI group we found that with older age at assessment, patients made fewer errors, were more accurate, more efficient and goal directed, were better at analyzing the situation and environment, and adjusting their behavior according to the task rules and social norms. In the HC group, we also found that younger children were more prone to omit steps or ingredients. However, there was no significant association between total errors on CCT and age at assessment. Taken together, we believe that our study supports previous findings that the CCT is sensitive to age-related differences.

### Convergent Validity

The convergent validity of the Norwegian version of CCT is supported by: (1) the significant association with performance-based tests assessing the EF domains of Cognitive flexibility and Working memory in the pABI group; (2) significant association with the ecologically sensitive BADS-C battery for both groups; and (3) significant association with teacher reported executive dysfunction on the BRIEF and DEX-C in the pABI group.

In the present study, a higher total number of errors on CCT was related to poorer ability to accurately switch between task sets (Cognitive flexibility), and poorer ability to add relevant and omit non-relevant information from working memory (Working memory). Furthermore, the error types that differentiated best between the pABI group and HCs were errors that could be classified within the same construct of Cognitive flexibility and Working memory. This supports the developmental model of EF proposed by Diamond, arguing that EF could to some extent be divided into separable EF processes. Chevignard et al. (2010) detected in their study an association between CCT and performance on verbal fluency task, a test that could be argued to measure aspects of the EF process of Working memory in Diamond’s model (Diamond, 2006; Shao et al., 2014). However, previous studies have found no other association between CCT and neuropsychological tests assessing Cognitive flexibility or Working memory (Chevignard et al., 2010; Fogel et al., 2020).

Contrary to our hypothesis, no significant associations were observed between the performance on CCT and the composite scores of Inhibition. However, we did find that a higher number of errors on CCT were associated with poorer performance on CWIT Inhibition and there was a trend toward an association between CCT and CPT-III Commissions. This suggests that to some extent, performance on CCT relies on the ability to resist making automatic responses and control actions, as well as ignore distractions. Furthermore, we did not find any association between performance on CCT and tests assessing Planning. This is in contrast to the study by Chevignard et al. (2010), where they found a significant association between CCT and a planning test. We may speculate that the illustrated checklist and the step-by-step recipe may provide the participants help with structuring and planning the task, and thus reduce the demands on their planning abilities when assessed with the CCT. However, some argue that functions such as updating/working memory, inhibition, and shifting/cognitive flexibility need to operate in an integrative manner in order to solve planning-related tasks such as the Tower of London (Miyake and Friedman, 2012). As both planning and inhibition are proposed to be functions loading on a general or common EF factor (Fleming et al., 2016), the lack of strong association between these measures may indicate that the CCT does not assess the general, unitary dimensions of EF. However, as factor models of EF suggests only two separable EFs within middle childhood and adolescence (Karr et al., 2018), an association for a multicomponent task such as CCT to more than two separable EFs might not be expected.

The associations between the CCT and the performance-based tests ranged from small to moderate, which is in accordance with previous findings from both children (Chevignard et al., 2009, 2010) and adult (Chevignard et al., 2008; Hendry et al., 2016) populations. The difference in administration with regard to structure and environmental control may also contribute to the lack of strong associations. Furthermore, the CCT and neuropsychological tests may also be considered to assess functions on different (functional) levels (i.e., ICF levels). In addition, a complex, multicomponent and open-ended task such as the CCT probably enlists several EF skills in order to perform well. While the test aims to tap into distinct EFs such as cognitive flexibility and working memory (Diamond, 2006, 2014), we cannot rule out the possibility that it also captures integrative metacognitive functions (Stuss and Knight, 2013), an underlying common EF function (Friedman and Miyake, 2017), or also relies on other cognitive functions such as processing speed. The inconsistent findings and the lack of strong associations between performance-based EF tests and complex, multicomponent, open-ended measures such as CCT may perhaps be best understood within such a framework (Chaytor and Schmitter-Edgecombe, 2003; Chevignard et al., 2009, 2010; Roy et al., 2015). This suggests that clear, strong associations with performance-based tests may not be expected.

Our study is the first to evaluate the relationship between performance on the CCT and all the BADS-C subtests. As both tests are perceived as ecologically sensitive, strong associations between these tests were expected. For both groups, there was a significant relationship between overall performance on the CCT and the BADS-C. On the subtest level, the results were more varied. In particular, the lack of association between the CCT and the Six Part Test in our study, which had previously been reported in studies of children (Chevignard et al., 2010) and adults (Chevignard et al., 2008), was unexpected. Although both tests are considered ecologically sensitive, the BADS-C is still a standardized task, with instructions, stringent scoring, behavioral constraints, and some degree of control over key variables, compared to CCT. This may lessen the burden on functions such as task-setting and problem-solving, and could partly explain the moderate associations. While performance-based tests are designed to focus mainly on one distinct EF function, ecological tests of EF and questionnaires often try to capture a wider range of EFs. This is more similar to daily life EF but also makes comparisons across assessment types and levels more difficult. Furthermore, as the CCT simulates daily life activities that are complex and open-ended, good performance seems to require several non-executive cognitive functions as well as EF (Burgess, 1997; Burgess et al., 1998), and this may confound the relationships between more ecologically sensitive tests.

Our study is one of few studies that have included teacher reports when evaluating content validity. Interestingly, we found that a higher number of errors on the CCT were associated with more problems related to EF in school settings, as reported by teachers. However, we found no association between the CCT and self- and parent reported EF, a finding in line with previous studies, showing no association to performance-based test nor the CCT (Chaytor and Schmitter-Edgecombe, 2003; Chaytor et al., 2006; Toplak et al., 2013). Even though the CCT simulates an everyday context (cooking) and has fewer behavioral constraints than performance-based tests, the CCT imposes some structure to the situation, and involves instructions and rules that are usually not found in the child’s daily life. Furthermore, the lack of convergence between self- and parent-report and performance-based tests may also be explained by different modes of assessment (Cicerone et al., 2006), and may be considered to assess different functional levels (Toplak et al., 2013). In addition, the accuracy of self- and by-proxy ratings may be influenced by several factors; reduced self-awareness following pABI (Prigatano and Altman, 1990; Hart et al., 2005), report biases such as social desirability, recall bias or context effects (Van de Mortel, 2008; Demetriou et al., 2015); emotional state, family distress and dynamics (Stokes et al., 2011), and the parent’s familiarity with their child’s everyday functioning (Norris and Tate, 2000). In contrast to parents, the teachers observe the child in a more structured environment, comparing the child to his or her peers in a high demanding, complex situation expecting efficient problem-solving on academic tasks as well as social interactions. As such, our study suggests that performance on a multicomponent task such as the CCT may be more similar to what is observed in the classroom than in the home setting.

Advancing current knowledge, we found a relationship between performance on CCT and overall function in everyday life, providing preliminary evidence that the complex goal-setting functions measured by CCT may have an important impact on the ability to perform well at school as well as in social relations with friends and family. The main goal of neuropsychological rehabilitation is to enable people with pABI to participate effectively in valued activities within the domains of psychological, social, leisure, vocational or everyday functioning (participation level within the ICF framework; Wilson et al., 2009). Consequently, it follows that to be able to identify areas of need and plan treatments, there is a need to advance outcome assessment through developing tools that better evaluate performance on the participation level (Wilson et al., 2009; Tate et al., 2014). While the cognitive demands in laboratory analogs of real-life tasks (e.g., the BADS-C; Emslie et al., 2003) may have a higher resemblance (verisimilitude) to those in the everyday environment than standardized neuropsychological tests (Manchester et al., 2004; Wood and Liossi, 2006; Lewis et al., 2011), they still imperfectly replicate real-life situations (i.e., performance), potentially masking application of strategies such as those taught in cognitive rehabilitation (Chaytor et al., 2006). Our findings suggest that the CCT may be a promising tool for evaluating performance at the participation level. In addition, the CCT provides a more detailed analysis of behavior with regard to the child’s problem-solving behavior, task-setting, task-shifting, and online control. This analysis could be used to develop treatment strategies and better confidence in results evaluating the effects of rehabilitation interventions.

### Divergent Validity

A high divergent validity of CCT is supported by the lack of associations between CCT and tests of Verbal IQ, Attention and Learning, and Memory, suggesting that CCT is able to target functions within the EF domain without triggering non-executive cognitive functions. Nevertheless, the presence of the illustrated check-list and the recipe book may lessen the demand on memory functions and may contribute further to the lack of associations between CCT and Learning and Memory. However, as the functions of working memory, attention, and learning in real life often work together and often depend on EF, caution should be applied when contrasting them. One of the major obstacles to capturing EF by any method of assessment, is the problem of “task impurity” (Burgess, 1997; Burgess et al., 1998), arguing that tasks that are believed to measure EF often also trigger cognitive processes from many non-executive cognitive systems (Hughes and Graham, 2002; Jurado and Rosselli, 2007), including visual intelligence. Visual intelligence and processing speed were related to performance on the CCT, which corroborate previous findings (Chevignard et al., 2010). Processing speed is a non-executive cognitive function that supports many other non-executive and executive skills, including the development of general intelligence and working memory (Fry and Hale, 2000; Palmer and Leigh, 2009), which may partly explain the association to performance on CCT. Furthermore, processing speed and intelligence are skills that have been shown to be related to the concept of EF (Duncan et al., 1996; Wood and Liossi, 2007), although other studies report no consistent association between EF and intelligence (Ardila et al., 2000). In particular tasks of visual intelligence often includes elements of planning, cognitive flexibility, and the ability to inhibit impulses, making these tasks more sensitive to EF impairments.

### Limitations of the Study

Our main aim was to evaluate the psychometric properties of the Norwegian version of the CCT in children and adolescents with pABI. As we excluded children and adolescents with diagnosed learning disabilities and neuropsychiatric diseases both among the children with pABI and the HCs, we cannot rule out the possibility of having included participants that did not meet inclusion/exclusion criteria due to unreported or undiagnosed difficulties. However, the children with pABI were all able to follow mainstream school prior to injury/diagnosis, and the HCs reported no problems at school during the recruitment process. Moreover, participants were recruited on the basis of different types of pABI, making it difficult to measure and compare injury or insult severity across all types of pABI. Only the pABI group underwent the full test panel and only a limited number of measures were available for comparisons across the whole sample. This study is based on the baseline assessment in an RCT, and power analysis was as such only performed in relation to the detection of effects in the clinical trial. A power analysis for the aims of the present study was therefore not calculated a-priori. Although our sample is relatively large, we did not have sufficient power to analyze and compare performance across the different types of pABI, which should be addressed in future studies. Likewise, our sample size did not allow for further exploration between performance on CCT and age at injury, something that would be interesting to address in future studies. In addition, different assessors and locations for assessment may have introduced potential errors in measurements. To prevent this, all assessors were trained and provided with a SOP to reduce variance. The good inter-rater reliability is also reassuring with regard to concerns about errors of measurement. Furthermore, we cannot rule out potential sub-optimal performance caused by cognitive fatigue on tests administered late during the long test days—even though measures such as randomization of test presentation in blocks and breaks were included to reduce some bias. We considered results with an alpha level between 0.01 and 0.05 as trends toward significance, and when including a high number of comparisons, this may have inflated Type 1 errors. Thus, p-values in this interval should be interpreted with caution.

### Conclusion

The Norwegian adaptation of CCT demonstrated good psychometric properties in the largest sample assessing EF with CCT among children and adolescents with pABI to date. Of note, the CCT demonstrated good reliability, in addition to acceptable convergent validity on all levels of performance-based EF, except for planning. Children with pABI perform worse on the CCT compared to typically developing children, and the CCT is sensitive to the developmental trajectory of EF from childhood throughout adolescence. While the CCT to some extent identified group membership, the sensitivity and specificity were overall classified as poor. Performance on the CCT was significantly associated with teacher-report, but not self- and parent report of the questionnaires. Interestingly, performance on the CCT was related to the overall level of disability and function, suggesting the performance relies on cognitive functions also important for activity in everyday life and participation in the society. Furthermore, the CCT has the potential to advance the assessment of EF by providing a more accurate behavioral analysis of real-world performance and identifying deficits in the child’s problem-solving behavior, goal-setting, and cognitive control that could be used to develop treatment strategies and represent a more valid outcome measure of rehabilitation interventions. Finally, our results suggest that processing speed and visual IQ, in addition to EF, are important for CCT performance. Nevertheless, further studies with larger sample sizes are needed to evaluate function across the different types of pABI. Furthermore, future studies should investigate predictors of task performance, as well as evaluate the ability of the CCT to detect improvement in EF over time.

## Data Availability Statement

The datasets generated and analyzed during the current study are not publicly available due to ethical standards for the treatment of patient data, but are available from the corresponding author on reasonable request.

## Ethics Statement

The studies involving human participants were reviewed and approved by Regional Committees for Medical and Health Research Ethics, Norway (2017/772/REK). Written informed consent to participate in this study was provided by the participants’ legal guardian/next of kin for participants under 16 years of age, or by the participant themselves if they were 16 years or older.

## Author Contributions

TF, SA, MC, AB, RH, KR, TR, and JS have developed the study protocol for this study. TF, AB, and JS conceived the original idea for the study design, and together with MC and SA selected outcome measures. TF, JS, and RH have been involved in the translational process of the outcome measures. TF, GJ, and AB have adapted the CCT material. TF has been responsible for the recruiting process, training of assessors, developing the Norwegian manual for CCT, and developing and inserting data into the webCRF. GJ has contributed to the recruiting process, developing the manual for CCT, assessed the participants, conducted the IRR, and inserted data into the webCRF. Finally, all authors have agreed to be accountable for all aspects of the work. All authors contributed to the article and approved the submitted version.

## Conflict of Interest

The authors declare that the research was conducted in the absence of any commercial or financial relationships that could be construed as a potential conflict of interest.

## Publisher’s Note

All claims expressed in this article are solely those of the authors and do not necessarily represent those of their affiliated organizations, or those of the publisher, the editors and the reviewers. Any product that may be evaluated in this article, or claim that may be made by its manufacturer, is not guaranteed or endorsed by the publisher.
